# Expeller Barrel Dry Heat and Moist Heat Pressure Duration Induce Changes in Canola Meal Protein for Ruminant Utilisation

**DOI:** 10.3390/ani8090147

**Published:** 2018-08-21

**Authors:** Rebecca Heim, Gaye Krebs

**Affiliations:** 1 ARC Industrial Transformation Training Centre for Functional Grains, Graham Centre for Agriculture and Innovation, Charles Sturt University, Wagga 2678, Australia; 2 School of Animal and Veterinary Sciences, Charles Sturt University, Wagga 2678, Australia; gkrebs@csu.edu.au

**Keywords:** ruminal, canola, cold press, cattle, moist heat, solubility

## Abstract

**Simple Summary:**

Canola meal, a by-product of oil production from canola seed, is a source of protein commonly incorporated into dairy and feedlot rations. Processing conditions and pressure treatments can alter the quality of protein in canola meal. In this study, the impact of expeller dry heat and moist heat pressure duration time on general nutritional properties, in vitro protein degradability, Maillard reaction product formation, and molecular and microscopic structural characteristics of canola meal were investigated. Increased dry heat temperature rapidly increased digestible protein and non-protein nitrogen content, and constricted amide II secondary structure. Increased moist heat pressure treatment duration promoted browning, and the conversion of protein to more intermediately and slowly degradable forms. Dry heat and moist heat pressure affected meal protein solubility and protein and lipid-related functional groups. Moist heat pressure fragmented canola meal into enzyme-resistant aggregates with crevices containing oil bodies. Induced changes may impact the supply of protein and amino acids and subsequently the yield and composition (protein and lipid) of milk produced by dairy cows. These findings benefit producers of canola meal by further describing the effects of processing and treatment conditions on protein characteristics, particularly those which affect the production potential of ruminants fed canola meal as a source of protein.

**Abstract:**

To improve the protein nutritional quality of canola (*Brassica napus* L.) meal, further investigation of the effects of processing conditions and post-production treatments is desirable. The impact of barrel dry heat temperature (20 °C (cold press) and 100 °C (expeller)) and moist heat pressure (MHP) duration time on general nutritional properties, Maillard reaction product (MRP) formation, in vitro protein degradability, and molecular and microscopic structural characteristics of canola meals were investigated. Increased MHP duration reduced (*p* < 0.05) dry matter, soluble protein, rapidly degradable protein, yellowness (early MRP), whiteness (late MRPs), absorbance at 294 nm (intermediate MRPs), and amide I; and increased (*p* < 0.05) non-protein N, neutral detergent fibre, neutral detergent insoluble crude protein (CP), intermediately and slowly degradable protein, in vitro effective CP degradability, redness, degree of colour change, and browning. Increased dry heat temperature reduced (*p* < 0.01) CP and rapidly degradable protein, constricted amide II, reduced (*p* < 0.05) protein solubility in 0.5% KOH and increased (*p* < 0.05) acid-detergent fibre and intermediate MRPs. Browning index and redness exhibited potential as rapid indicators of effective CP degradability and soluble protein, respectively. Dry heat and MHP altered (*p* < 0.05) lipid-related functional groups. Dry heat affected napin solubility, and MHP altered cruciferin and napin solubility. Application of MHP induced the formation of proteolysis-resistant protein aggregates with crevices containing oil bodies. Induced changes may impact the supply of proteins and amino acids and subsequently the yield and composition (protein and lipid) of milk produced by dairy cows.

## 1. Introduction

Global demand for animal-derived protein is projected to double by 2050 [1], in turn increasing requirement for more animal feed. Canola (*Brassica napus* L.) meal is a readily available by-product of canola oil production. Due to its desirable amino acid (AA) profile and digestibility, canola meal is commonly utilised as a protein supplement in dairy cattle [2,3] and feedlot [4,5] rations. To generate canola meal, solvent extraction and mechanical (for example, cold press, expeller, and extrusion) technologies are applied. The protein content of the meal differs depending on the oil extraction method used [6,7]. During cold press extraction, seeds are mechanically pressed at low heat (≤65 °C) from frictional forces within the expeller barrel to produce canola meal with 11–13% lipid [7,8]. Expelling utilises moderate temperatures (95–135 °C) to generate canola meal with 15–18% moisture and 8–15% lipid [7,8,9]. Expeller heat may increase rumen undegraded crude protein (RUP) by establishing cross-linkages among and within peptide chains, and to carbohydrates [10].

To reduce ruminal degradation, and in turn increase the post-ruminal supply of canola protein, AA studies have evaluated different chemical and heat treatments. For instance, dry heating (125 °C, 10 min) of canola meal was reported to decrease in situ rumen crude protein (CP) disappearance without compromising intestinal digestibility in ruminants [11]. Dry heating (125 °C, 20 min) of expeller canola meal was reported to reduce rumen CP degradability and, when fed to primiparous cows, increase milk production [12]. Alternatively, treatment with moist heat pressure (MHP, autoclaving) involves heating meals with steam under pressure. Application of canola meal with MHP (117 kPa, 127 °C, 15 or 30 min) induced partial protein denaturation and decreased ruminal protein degradability [13], and was later reported to increase the post-ruminal supply of AA for digestion in the small intestine [14]. When diets of dairy cows were supplemented with MHP-treated cold-pressed rapeseed (*Brassica rapa* L. *oleifera* subv. *annua*) cake, milk yields increased relative to an untreated rapeseed meal [15].

Throughout processing, protein digestibility may be reduced by the formation of compounds that inhibit digestive enzymes or by the modification of the protein molecule, for example, blocking of active AA side-chains, or the formation of crosslinks [16]. During the oil extraction process, the heat-damaged protein formed within the meal from the Maillard reaction is of particular concern for ruminant nutritionists as it contributes to RUP levels without providing nutritional benefits [17]. Traditionally, acid detergent insoluble N (ADIN) was utilised to monitor heat-damage protein; however, it is theorised ADIN analysis does not quantitatively account for all Maillard reaction products (MRPs) [18]. Consequently, it is of interest to quantify the production of MRPs during processing of canola meal utilising other established techniques, for instance, pH, UV-Vis absorbance [19], colorimetry, and gel-electrophoresis [20].

To monitor changes in ruminal degradability, economical, high-throughput and non-invasive alternatives to in vivo, in situ and in vitro ruminal fluid procedures have been established. These include for example proteolytic assays, mathematical modelling [21], near-infrared reflectance spectroscopy [22], and molecular spectroscopy [23,24,25,26]. Utilising attenuated total reflectance–Fourier transform infrared (ATR-FTIR) molecular spectroscopy, protein structure characteristics of canola meal were found to strongly correlate with in situ ruminal degradable CP [23,24]. Furthermore, ATR-FTIR molecular spectroscopy and synchrotron-radiation-based microspectroscopy were utilised to characterise the impact of dry heat and MHP on the protein structure for canola seed [25] and canola seed tissue [26], respectively. Alterations in microscopic structure resulting from solvent extraction processing were reported for rapeseed meal [27]. Confocal laser scanning microscopy (CLSM) [28], and scanning electron microscopy (SEM) [28,29] techniques have been applied to investigate the resistance of protein structure to enzymatic degradation in soybean meal and dried-distillers grains.

To improve the protein value of canola meal for ruminants, the objectives of this study were to examine the effects of low (i.e., cold press, 20 °C) and high (i.e., expeller, 100 °C) barrel dry heat processing conditions and MHP treatment duration time on general nutritional properties, protein degradability, MRP formation, and the molecular and microscopic structural characteristics of canola meal.

## 2. Materials and Methods

### 2.1. Canola Meal and Suspension Preparation

#### 2.1.1. Canola

Commercial bulk-handling canola seed was provided by MSM Milling (Manildra, NSW, Australia). The (~5 kg) heterogeneous seed lot was stored at room temperature (RT, ~21 °C), in an air-tight hessian polypropylene bag within a dark and dry cupboard.

#### 2.1.2. Barrel Dry Heat and Moist Heat Pressure of Canola

To prepare canola meals, seed (~240 g) was passed separately through a primed bench-top screw-press expeller (Model DSZYJ-200A/B (Taizhou Dengshang Mechanical Electrical Co. Ltd, Zhejiang, China), 220 V, 50 Hz, 50 rpm) at a barrel dry heat temperature of either 20 °C (RT, cold-press) or a pre-heated temperature of 100 °C (expeller); this was then repeated two more times (*n* = 2 × 3, 6). The meals were individually ground in an electric mill (Breville Grinder, CG2B) and passed through a 1-mm sieve. The MHP treatment was completed by placing each meal (40 g) in a separate flat rectangle polypropylene container and autoclaving using a steriliser (Atherton Centenary Series, Melbourne, Australia) set on the Hard Goods Dry Cycle No. 1.1, for 0, 3, 6, 9 or 12 min (192 kPa, 120 °C). For each triplicate meal, an independent sterilising cycle was performed (*n* = 6 × 5, 30). The meals were stored in the dark at RT.

#### 2.1.3. Preparation of Canola Meal Suspensions and Pellets

The meals were ground (<5 µM) by placing 5 g of meal in a stainless-steel screw-top grinding jar (50 mL) with a ⌀ 25-mm grinding ball. The jar was positioned in a mill (MM301, Retsch, GmbH, Hann, Germany) and shaken for 30 s (frequency 20 per s) followed by a 15-s rest, thrice. To generate suspensions, ground meal (200 mg) was added to deionised H_2_O (10 mL) and shaken for 30 min using a Multi Reax (Heidolph Instruments GmbH, Schwabach, Germany) set at 10. The suspensions were stored in the dark at 4 °C. To prepare circular pellets, canola meal (~0.2 mg) was pressed at 100 bar utilising an Hydraulic Press (Enerpac, Menomonee Falls, WI, USA).

### 2.2. General Nutritional Characteristics

The meals were analysed for dry matter (DM) (AOAC 930.15 (Association of Official Agricultural Chemists)), lipid (AOAC 992.06), CP (6.25 × N) by Leco Dumas N combustion (AOAC 992.23), and carbohydrate [30]. To determine the quantity of carbohydrate (%DM), each meal suspension (40 µL) was added separately to deionised H_2_O (10 µL), concentrated sulphuric acid (150 µL), and 5% phenol in deionised H_2_O (30 µL) in a clear flat bottom non-absorbent 96 F Microwell microplate (Nunc #269620) (Thermo Scientific, Waltham, MA, USA). The plate was incubated for 5 min at 90 °C in a shallow water bath, rested for 5 min at RT, wiped dry, placed in a CLARIOstar 5.20 R5 microplate reader (BMG LABTECH, Ortenberg, Germany), shaken at 500 rpm for 10 s, and measured for absorbance at 490 nm. Values were corrected by deducting an average of blank measurements. A standard curve (0–10 nmol) was established using a 1 M stock solution of D-mannose (Sigma, St Louis, MO, USA) prepared in deionised H_2_O.

### 2.3. Protein Solubility and Fractionation

The meals were analysed for soluble protein [31] and solubility in 0.5% KOH [32]. The latter was performed by stirring samples of the meal (5 g) in 0.5% KOH (33.3 mL) for 20 min, centrifuging at 1250× g for 10 min, and quantifying the protein in the supernatant by Leco Dumas N combustion. The meals were analysed in duplicate (*n* = 4 × 5, 20) for non-protein N (NPN, tungstic acid [31]), acid detergent fibre (ADF), neutral detergent fibre (NDF), ADIN and neutral detergent insoluble N (NDIN) by the Australian Oil Reference Laboratory (Department of Primary Industries, NSW, Australia). Results were utilised to calculate true protein: %, CP − NPN. The meals were partitioned into protein fractions based on characteristics of degradability according to the Cornell Net Carbohydrate and Protein System (CNCPS) as described [33]. Using CNCPS, Fraction A is NPN, Fraction B is degradable protein containing B1 (soluble protein, rapidly soluble in the rumen), B2 (intermediate degradation, Total CP − (A + B1 + B3 + C)), and B3 (slowly degraded in the rumen, NDIN − ADIN), and Fraction C is undegradable protein (ADICP).

### 2.4. In Vitro Effective Protein Degradability

All meals were analysed for in vitro effective CP degradability (i.e., estimated RUP) utilising the in vitro proteolysis procedure by Krishnamoorthy, et al. [34] validated in vivo in lactating dairy cattle (*r*^2^ = 0.61). The meal (0.5 g) was weighed into a 125-mL Erlenmeyer flask and incubated at 39 °C for 1 h in 40 mL borate-phosphate (BP) buffer (pH 8.0). *Streptomyces griseus* protease (Type XIV 5.4 U per mg protein, Sigma P-5147) solution (0.33 U per mL, 10 mL BP-buffer) was added, and the meal was incubated at 39 °C for 18 h. The residue was collected on quantitative filter paper (22 µm pore, No. 541, Whatman, Maidstone, UK), rinsed with distilled H_2_O and air-dried overnight. All flasks were placed on ice to suspend proteolytic activity before filtering. Residual CP was determined by combusting the whole filter paper by Leco Dumas N combustion:

(1)  Effective CP degradability (% of CP ) = (CP − undegraded CP)/ CP × 100

### 2.5. In Vitro Intestinal Digestion of Protein in Ruminants

The meals were analysed in duplicate for in vitro intestinal digestion of CP utilising the HCl-pepsin pre-digestion procedure of Calsamiglia and Stern [35] validated in vivo (*r* = 0.91). In a 50-mL Falcon tube sample (15 mg CP) was suspended in 10 mL pH 1.9, 0.1 N HCl solution of 1 g per L pepsin (Sigma P-7012), vortexed, and incubated at 38 °C for 1 h in a shaking H_2_O bath. Pancreatin solution (13.5 mL: 0.5 M KH_2_PO_4_ pH 7.8 containing 3 g per L pancreatin, Sigma P-7545) and 1 N NaOH (0.5 mL) was added, and the tube was vortexed, incubated at 38 °C for 24 h in a shaking H_2_O bath, vortexing every ~8 h. To cease the reaction, trichloroacetic acid (TCA) (3 mL) was added, the tube was vortexed, rested (15 min), and then centrifuged (10,000× *g*, 15 min). The supernatant was analysed for soluble N, as described. Results were utilised to calculate %pepsin–pancreatin digestion of protein:

(2)  %IVCPD = TCA − soluble N/ initial N ×100 

### 2.6. Measurement of Maillard Reaction Products

#### 2.6.1. Measurement of Colour

To monitor Maillard reaction product formation, meals were separately placed in a lidded cuvette then colour was measured with a Chroma Meter CR-300 colorimeter (Minolta CO., Osaka, Japan), using the CIE-Lab tristimulus system, calibrated with a white tile and a D-65 illuminant source. The a* (red-green), b* (yellow-blue) and L* (white-black), degree of colour change (∆*E*), and browning index (BI) were calculated as previously described [36].

#### 2.6.2. Measurement of pH

The meal suspensions were monitored for pH by magnetic stirring using a PHM 93 Reference pH meter (Radiometer, Copenhagen, Denmark) calibrated with buffer solutions at pH 4 and 7.

#### 2.6.3. Determination of UV-Vis Absorbance at 294 nm

The meal suspensions were analysed for UV-Vis absorbance utilising an adapted procedure [19]. The meal suspension (20 µL) was added to deionised H_2_O (80 µL) in a clear flat bottom non-absorbent 96 F 400 µL Microwell microplate (Nunc #269620), and UV-Vis Abs_294nm_ was measured utilising a CLARIOstar 5.20 R5 microplate reader (BMG Labtech, Offenburg, Germany).

### 2.7. Measurement of Structural Changes

#### 2.7.1. Measurement of Surface Hydrophobicity

The meals were analysed in duplicate for surface hydrophobicity (*So*) with fluorescence probes [37]. Under darkened conditions, an aliquot of canola meal suspension was made up to a final volume of 300 µL using 0.01 M sodium tetraborate solution pH 6 in a 400 µL 96-well microplate (Nunc™ F96 MicroWell™ Black Polystyrene 237105). To each well, 1 µL of 1-anilino-8-naphthalene sulfonate (ANS, Fluka 10419) reagent was added. The plate was incubated at 25 °C for 2 min, then shaken at 500 rpm for 10 s in a CLARIOstar 5.20 R5 microplate reader. Fluorescence intensity was measured at an excitation maximum of 390–405 nm and an emission maximum of 470–500 nm. Values were corrected by deducting an average of blank measurements. To determine sample *So*, a 0–200 µg per µL standard curve was established using a 1 M stock solution of bovine serum albumin (Sigma) prepared in 0.01 M sodium tetraborate solution and stored in the dark at 4 °C.

#### 2.7.2. ATR-FTIR Sample Preparation, Data, and Collection Analysis

The molecular spectral data of canola meal pellets were generated by ATR-FTIR 8400S (Shimadzu Corp., Kyoto, Japan) with a single reflection plate, flat tip, and constant pressure (530 psi) in absorbance mode (40 scan runs, 4 cm^−1^, Happ-Genzel apodisation, mid-IR, approximately (ca.) 4000–600 cm^−1^). The data were collected utilising IR Solution software, baseline corrected [38], and total peak normalised. The lipid [39], and protein [25] functional groups were identified. Briefly, the IR total protein fingerprint region ca. 1714–1480 cm^−1^ included amide I (AI, ca. 1714–1571 cm^−1^), amide II (AII, ca. 1572–1480 cm^−1^), α-helix (peak centre height at ca. 1652 cm^−1^ with the baseline of ca. 1714–1480 cm^−1^), and β-sheet (peak centre height at ca. 1630 cm^−1^ with a baseline of ca. 1714–1480 cm^−1^). Lipid regions included the lipid carbonyl C=O ester stretching band (LCCE, baseline ca. 1789–1701 cm^−1^ with peak ca. 1744 cm^−1^), CH_3_ asymmetric (CH_3A_ ca. 2988–2951 cm^−1^ with peak centre at ca. 2955 cm^−1^), CH_2_ asymmetric (CH_2A_ ca. 2951–2882 cm^−1^ with peak centre at ca. 2922 cm^−1^), CH_3_ symmetric (CH_3S,_ ca. 2882–2868 cm^−1^ with peak centre at ca. 2872 cm^−1^) and CH_2_ symmetric (CH_2S_ ca. 2868–2790 cm^−1^ with peak centre at ca. 2852 cm^−1^).

#### 2.7.3. Gel Electrophoresis of Canola Meal Protein Profiles

##### Sodium Dodecyl Sulphate-Polyacrylamide Gel Electrophoresis

The polypeptide banding profiles of duplicate canola meal samples were visualised utilising an adapted SDS-PAGE procedure [40] as follows; the sample (~10 mg CP) was dissolved in sample buffer (1 mL: 11.25 mM tris-HCl, pH 8.5, 3.6% SDS, 18% glycerol, and 0.0025% bromophenol blue), and heated at 85 °C for 10 min. For reducing conditions, 50 mM dithiothreitol (DTT) was added to the sample buffer. The protein sample (30 µg of CP per well) and standard marker (5 µL, Novex Mark 12, Invitrogen, Mulgrave, VIC, Australia) were loaded onto a NuPAGE gradient precast gel (4–12% gradient) bis-tris (10 × 10 cm^2^) in a Novex Xcell mini cell system (Invitrogen, Mulgrave, VIC, Australia). Electrophoresis was performed at 80 V for 75 min, followed by 90 V for 75 min in running buffer (50 mM methysulfonic acid, 50 mM tris base, 0.1% SDS, 1 mM ethylenediaminetetraacetic acid, pH 7.3). The polypeptide bands were visualised by incubating the gel in Coomassie Brilliant Blue R-250 solution (0.1% in 40% methanol, 10% acetic acid) for 25 min, and de-stained (10% ethanol and 7.5% acetic acid) on an orbital shaker at RT overnight.

##### Native Gel Electrophoresis

The native protein profiles of duplicate canola meal samples were visualised by gel-electrophoresis following manufacturer’s instructions. In brief, sample (~10 µg CP per µL) was added to 2.5 µL NativePAGE^TM^ sample buffer (4×), 1 µL NativePAGE^TM^ 5% G-250 sample additive, and made to 10 µL with deionised H_2_O. The sample and NativeMark^TM^ unstained protein standard (5 µL, LC0725, Invitrogen, Mulgrave, VIC, Australia) were loaded onto a NativePAGE^TM^ 4–16% gradient precast bis-tris (10 × 10 cm^2^) gel in a Novex Xcell mini cell system (Invitrogen, Mulgrave, VIC, Australia). The running buffer contained 50 mM BisTris, 50 mM tricine, pH 6.8, and the sample buffer contained 50 mM BisTris, 6 N HCl, 50 mM NaCl, 10% w/v glycerol, and 0.001% Ponceau S, pH 7.2. The upper (inner) buffer chamber contained cathode buffer (200 mL: 10 mL NativePAGE^TM^ running buffer 20×, 10 mL NativePAGE^TM^ cathode additive 20×) and the lower (outer) buffer chamber contained anode buffer (600 mL: 50 mL NativePAGE^TM^ running buffer 20×, 950 mL deionised H_2_O). Electrophoresis was performed at 150 V for 110 min. The native proteins were visualised by incubating the gel in 40% methanol and 10% acetic acid for 25 min, Coomassie Brilliant Blue R-250 solution (0.02% in 30% methanol and 10% acetic acid) for 25 min, and 8% acetic acid on an orbital shaker at RT overnight.

#### 2.7.4. Confocal Laser Scanning Microscopy

The microscopic structure of duplicate canola meal and in vitro proteolytic digested CP residues were analysed at RT utilising a TCS SP5 confocal laser-scanning microscope (CLSM, Leica Microsystems, Wetzlar, Germany) fitted with a 20× oil immersion objective. The meal (100 mg) was fluorescently labelled in Fast Green FCF (1 drop, 0.4% in H_2_O) and Nile blue (1 drop, 0.5% in H_2_O) dyes, to stain for protein and lipid, then excited at 633 and 488 nm, and reflected emitted light was collected at 662–744 and 520–626 nm with HeNe and argon lasers, respectively.

#### 2.7.5. Scanning Electron Microscopy

The duplicate canola meal and in vitro proteolytic digested CP residues were adhered to aluminium sample holders using double-sided smoothest carbon tabs (ProSciTech Pty Ltd, Kirwan QLD, Australia). The samples were imaged in a S4300 SE/N variable pressure scanning electron microscope (SEM) (Hitachi, Tarrytown, NY, USA). The environmental secondary electron detector was used with a pressure of 50 Pa, accelerating voltage of 20 kV at RT and a working distance of 15 mm.

### 2.8. Statistical Analysis

Statistical analyses of data were performed using the statistical software OriginLab v 95E (Origin, Northampton, MA, USA). To establish differences, the one-way ANOVA mathematical model used for analysis was:

(3) $$\;Y_{ij}\;=\;\mu \;+\;T_{j}\;+\;e_{ij}\;$$

where Y*_ij_* is an observation on the dependent variable *ij*; µ is the population mean for the variable, and T*_j_* is the effect of treatment (*i* = MHP duration time and/or barrel dry heat temperature), as a fixed effect. The independent barrel runs at each temperature were experimental replications and e*_ij_* value is the random error associated with the observation *ij*. A post hoc Fisher’s least significant difference test was performed to determine the statistical significance of differences between individual means, declared at *p* < 0.05. Normal distribution was established by performing an Anderson-Darling test, *p* > 0.05. The Spearman correlation coefficient (*r_s_*) with a two-tailed test of significance (*p* < 0.05) was used to define strength and association of relationships between MHP duration time and dependent variables. Polynomial regression was performed to determine the coefficient of determination (*r*^2^), using the equation:

(4) $$\;Y_{i}\;=\;\beta_{0}\;+\;\beta_{1}x_{i}\;+\;\beta_{2}{x^{2}}_{i}\;+\;\varepsilon_{i}$$

## 3. Results

### 3.1. General Nutritional Characterisation of Canola Meals

The effects of barrel dry heat and MHP duration on general nutritional characteristics of canola meal are presented in Table 1 and Table A1. The CP content of the non-treatment cold-pressed meal (33.8%) was higher (*p* < 0.01) than that of expeller canola meal (33.2%). The CP content decreased (*p* < 0.05) with MHP durations (*r_s_* = −0.35, −0.36 for cold-pressed and expeller meals, respectively). The DM content decreased (*p* < 0.05) with MHP duration (*r_s_* = −0.74, −0.80 for cold-pressed and expeller meals, respectively) and was similar (*p* > 0.05) in non-treatment cold-pressed (92.8%) and expeller meals (93.1%). Lipid content remained similar between MHP durations in cold-pressed meals (*p* > 0.05) unlike expeller canola meals (*p* < 0.05). The lipid content did not vary (*p* > 0.05) between non-treatment cold-pressed (15.9%) and expeller (15.3%) meals. Carbohydrate content decreased (*p* < 0.05) with increasing MHP duration time in expeller meals (*r_s_* = −0.58), unlike in the cold-pressed meals (*r_s_* = −0.26). Carbohydrate content did not vary (*p* > 0.05) between the non-treatment cold-pressed (14.9% DM) and expeller (15.0% DM) meals. NPN, NDF, NDICP, and ADICP contents were similar (*p* > 0.05) between dry heat temperatures, whereas ADF was greater (*p* < 0.05) in non-treatment expeller (16.6%) as compared to cold-pressed (15.1%) meals (see Table 1 and Table A1). Overall, NPN, NDF, and NDICP (*r_s_* = 0.84, 0.82, 0.96) were strongly and positively (*p* < 0.05) associated with MHP duration in cold-pressed meals, unlike ADF and ADICP (*r_s_* = −0.12, 0.26) (*p* > 0.05).

### 3.2. Barrel Dry heat and MHP Duration Induced Changes in Protein in Canola Meal

#### 3.2.1. Protein Solubility

The effects of barrel dry heat and MHP duration on BP-buffer protein solubility (pH 6.7) and solubility in 0.5% KOH of canola meals are presented in Table 1 and Table A1. Protein solubility in BP buffer decreased (*p* < 0.01) with increasing MHP duration in cold-pressed (*r_s_* = −0.97) and expeller meals (*r_s_* = −0.95). Protein solubility in BP-buffer was lower (*p* < 0.05) in non-treatment expeller (69.0%) than cold-pressed (70.0%) meals. In cold-pressed meals, unlike expeller meals, 0.5% KOH solubility decreased (*p* < 0.05) with increasing MHP duration (*r_s_* = −0.98, −0.35). Protein solubility in 0.5% KOH was less (*p* < 0.05) in non-treatment expeller (34.7%) as compared to cold-pressed (55.1%) meals and decreased (*p* < 0.01) with increasing MHP duration (*r_s_* = −0.98, *r^2^* = 0.80).

#### 3.2.2. Protein Degradability

The effects of dry heat and MHP duration on effective CP degradability (ECPD; %CP) and in vitro CP digestibility (IVCPD) content of canola meals are presented in Table 1 and Table A1. The ECPD was positively associated with MHP duration (*r*^2^ = 0.97, *r_s_* = 0.97), and was similar (*p* > 0.05) in non-treatment cold-pressed (16.8) and expeller (17.3) meals. The IVCPD content of meals was unaffected (*p* > 0.05) by MHP duration and was similar (*p* > 0.05) in non-treatment cold-pressed (14.1%) and expeller (14.0%) meals.

#### 3.2.3. Protein Fractionation

Protein fractions B2, B3 and C were similar (*p* > 0.05) between dry heat temperatures, whereas A was less (*p* < 0.05) and B1 was greater (*p* < 0.01) in non-treatment cold-pressed (0.05, 69.8) meals as compared to expeller meals (0.05, 66.9), respectively (see Table 2). Overall, Fractions A, B2, and B3 (*r_s_* = 0.95, 0.87, 0.94) were positively and B1 (*r_s_* = −0.96) was negatively associated (*p* < 0.05) with MHP duration, except C (*r_s_* = 0.86) which was positively (*p* < 0.05) associated in cold-pressed meal only (see Table 2 and Table A2).

### 3.3. Barrel Dry Heat and MHP Induced Formation of Maillard Reaction Products

#### 3.3.1. Colour Development

Colour development as a function of dry heat and MHP duration in canola meals is presented in Table 3 and Table A2. As MHP duration increased the meals decreased (*p* < 0.01) in yellowness (b*, *r_s_* = −0.54, −0.90), decreased (*p* < 0.05) in whiteness (L*, *r_s_* = −0.64, −0.69), and increased (*p* < 0.01) in redness (a*, *r_s_* = 0.94, 0.90). Non-treatment expeller and cold-pressed canola meals were similar (*p* > 0.05) in whiteness (54.7 vs. 54.5), redness (1.80 vs. 1.56), and yellowness (17.3 vs. 17.0). The degree of colour change was positively associated (*r_s_* = 0.73, 0.88) and increased (*p* < 0.01) with MHP duration. As MHP duration increased the cold-pressed (*r_s_* = 0.95) and expeller canola meals (*r_s_* = 0.87) increased (*p* < 0.01) in BI. Non-treatment expeller (73.4) and cold-pressed (68.2) meals had similar (*p* > 0.05) BI values.

#### 3.3.2. Acidity

The pH was used as a measure of Maillard reaction-associated protein-sugar covalent bond formation in canola meal suspensions. As presented in Table 3 and Table A2, pH varied (*p* < 0.01) between MHP duration times, where acidity increased with MHP duration (*r*^2^ = 0.83, 0.67). The pH was similar (*p* > 0.05) in the expeller (6.40) and cold-pressed (6.39) suspensions.

#### 3.3.3. Intermediate Maillard Reaction Product Formation

UV-Vis Abs_294nm_ was used as a measure of intermediate-MRP formation with increasing MHP duration in canola meal suspensions, as presented in Table 3 and Table A2. Intermediate MRP content was dissimilar (*p* < 0.01) among MHP durations with negative (*r_s_* = −0.74, −0.63) associations of intermediate-MRP formation and MHP duration time observed. Intermediate MRP formation was increased (*p* < 0.05) in non-treatment expeller (1.93) compared to cold-pressed (1.61) meals.

### 3.4. Barrel Dry Heat and MHP Induced Changes in Protein Structure

The impacts of changing barrel dry heat and increasing MHP duration on surface hydrophobicity (*So*) in canola meal suspensions are presented in Table 4 and Table A2. *So* was similar (*p* > 0.05) between expeller (1.94) and cold-pressed (3.73) meal suspensions. There was a positive correlation (*r*^2^ = 0.72) between *So* and MHP duration in the expeller but not in the cold-pressed meal suspensions. In addition, *So* differed (*p* < 0.05) between MHP durations in the expeller but not the cold-pressed meal suspensions.

### 3.5. Barrel Dry Heat and MHP Induced Changes in Spectral Characteristics of Protein and Lipid Structure

**Figure A1:**
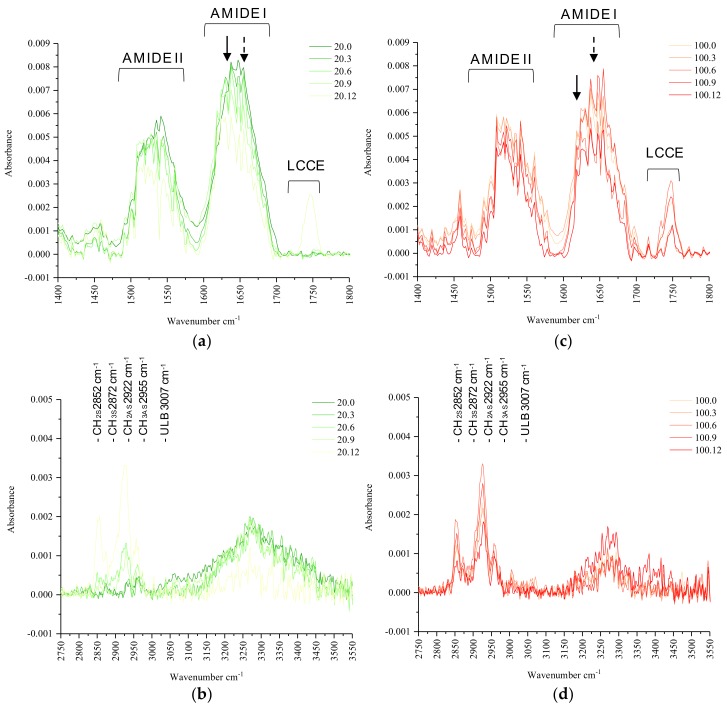
Total protein fingerprint region 1400–1800 cm^−1^ (**a**,**c**) and lipid spectra (**b**,**d**) of canola meals produced at alternate barrel dry heat temperatures (20 °C vs. 100 °C) without or with moist heat pressure at increasing durations (0, 3, 6, 9 or 12 min). The α-helix (dashed arrow, 1652 cm^−1^), β-sheet (arrow, 1630 cm^−1^), amide II (ca. 1572–1480 cm^−1^), amide I region (ca. 1714–1571 cm^−1^), and lipid carbonyl C=O ester stretching band (LCCE, ca. 1789–1701 cm^−1^), asymmetric CH_3_ (CH_3AS_ ca. 2988–2951 cm^−1^ with peak centre at ca. 2955 cm^−1^), asymmetric CH_2_ (CH_2AS_ ca. 2951–2882 cm^−1^ with peak centre at ca. 2922 cm^−1^), symmetric CH_3_ (CH_3S_ ca. 2882–2868 cm^−1^ with peak centre at ca. 2872 cm^−1^) and symmetric CH_2_ (CH_2S_ ca. 2868–2790 cm^−1^ with peak centre at ca. 2852 cm^−1^) functional groups in the attenuated total reflectance-Fourier transform infrared (ATR-FTIR) spectrum of canola meals. Spectra of a single representative replicate shown.

Infrared molecular spectroscopic characteristics (absorbed area intensity of the protein fingerprint region, and height intensities of α-helix, β-sheet, AI and AII, and their respective ratios) of the protein structure of canola meal processed with increasing MHP durations are presented in Table 4 (and Figure A1). In cold-pressed meal, but not expeller meal, MHP duration negatively (*p* < 0.05) correlated with AI (r_s_ = −0.53), α-helix (r_s_ = 0.79), and the ratio of α-helix-β-sheet (r_s_ = −0.90). In both cold-pressed and expeller meals, the AI region differed (*p* < 0.05) between MHP durations. Protein molecular regions were similar among barrel temperatures, except AII (0.271 vs. 0.299) was reduced (*p* < 0.01) in non-treatment expeller compared to cold-pressed canola meals.

Infrared molecular spectroscopic characteristics of lipid structure (absorbed height intensities of CH functional groups, and LCCE bands) of non-treatment cold-pressed and expeller meals treated at increasing durations with MHP are presented in Table 5 (and Figure A1). MHP duration did not (*p* > 0.05) induce structural changes in CH_3AS_ functional groups or ratios of CH_3_-CH_2_ asymmetric and symmetric functional groups. MHP was positively (*p* < 0.05) associated with and induced structural changes of the LCCE bands (*r_s_* = 0.56), CH_2AS_ (*r_s_* = 0.72), CH_3S_ (*r_s_* = 0.78), and CH_2S_ (*r_s_* = 0.79) in cold-pressed meals. Absorbance intensities of CH_2AS_, CH_3S_, and CH_2S_ were higher (*p* < 0.05) in non-treatment expeller than cold-pressed meals (0.002 vs. 7.34 × 10^−5^, 5.21 × 10^−4^ vs. 2.24 × 10^−4^, and 8.72 × 10^−4^ vs. −8.58 × 10^−5^_,_ respectively). The CH_3_-CH_2_ asymmetric ratio was similar (*p* > 0.05), and CH_3_-CH_2_ symmetric ratio was greater (*p* < 0.01) in non-treatment expeller than cold-pressed canola meals.

### 3.6. Barrel Dry Heat and MHP Induced Changes in the Protein Profile

**Figure 1:**
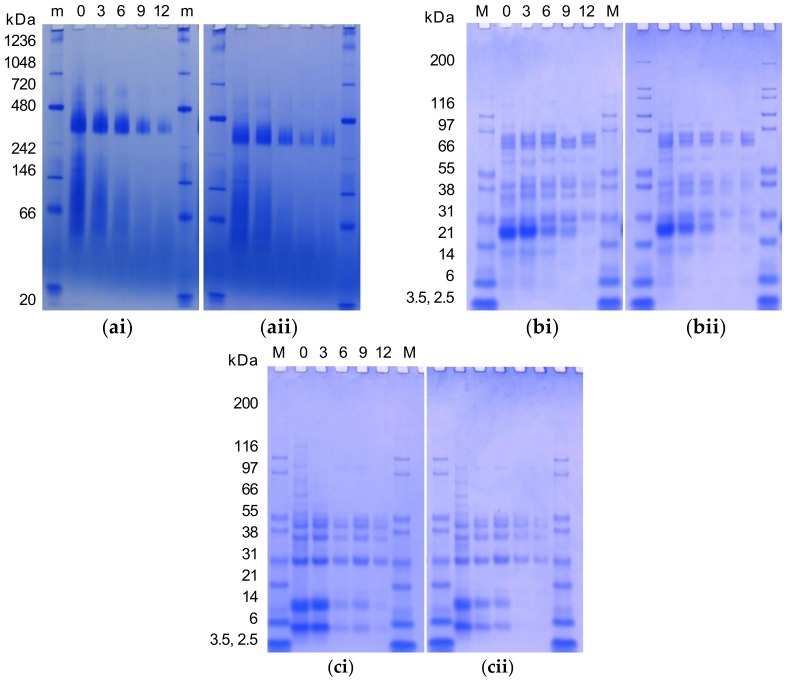
Native gel electrophoresis (**a**), and SDS-PAGE (non-reduced, (**b**)) and (reduced, (**c**)) water-soluble (pH 7) protein profile of cold-pressed (20 °C, (**i**)) and expeller (100 °C, (**ii**)) canola meal with moist heat pressure treatment for different durations (0, 3, 6, 9 or 12 min) revealed with Coomassie Blue Stain. Representative images presented. A 30 µg aliquot of each sample was loaded per well. A 5 µL aliquot of NativeMark™ Unstained Protein Standard (m) or Mark 12 Protein Standard (M) was loaded.

The gel electrophoresis analysis of water-soluble (pH 7) native, non-reduced, and reduced protein subunits of cold-pressed and expeller meals treated with increasing MHP durations are presented in Figure 1. Native conformation of water-soluble expeller and cold-pressed canola meal proteins consisted of a large 300–400 kDa protein band and protein smearing from 50 to 200 kDa. With increasing MHP duration the 300–400 kDa protein band varied in intensity. In non-reduced conditions, protein polypeptide bandings included: ~177, ~118, 73.5, 76.8, 41–55, 37.1, 28.5, 27.5, 26.7, 22.9, 21.1, 18.3, and 14 kDa. Under non-reducing conditions in water-soluble protein fractions, polypeptide banding intensity was reduced at 6 min of MHP duration. A 14 kDa polypeptide band was reduced at 12 min and 9 min of MHP duration in cold-pressed and expeller meals, respectively. Under reducing conditions in water-soluble protein fractions, polypeptide banding intensity noticeably reduced at 6 min and 3 min of MHP duration in cold-pressed and expeller meals, respectively. In both meal types, increasing MHP duration decreased the intensity of 4 and 9 kDa polypeptide bands.

### 3.7. Barrel Dry Heat and MHP Induced Changes in the Structural Organisation

**Figure 2:**
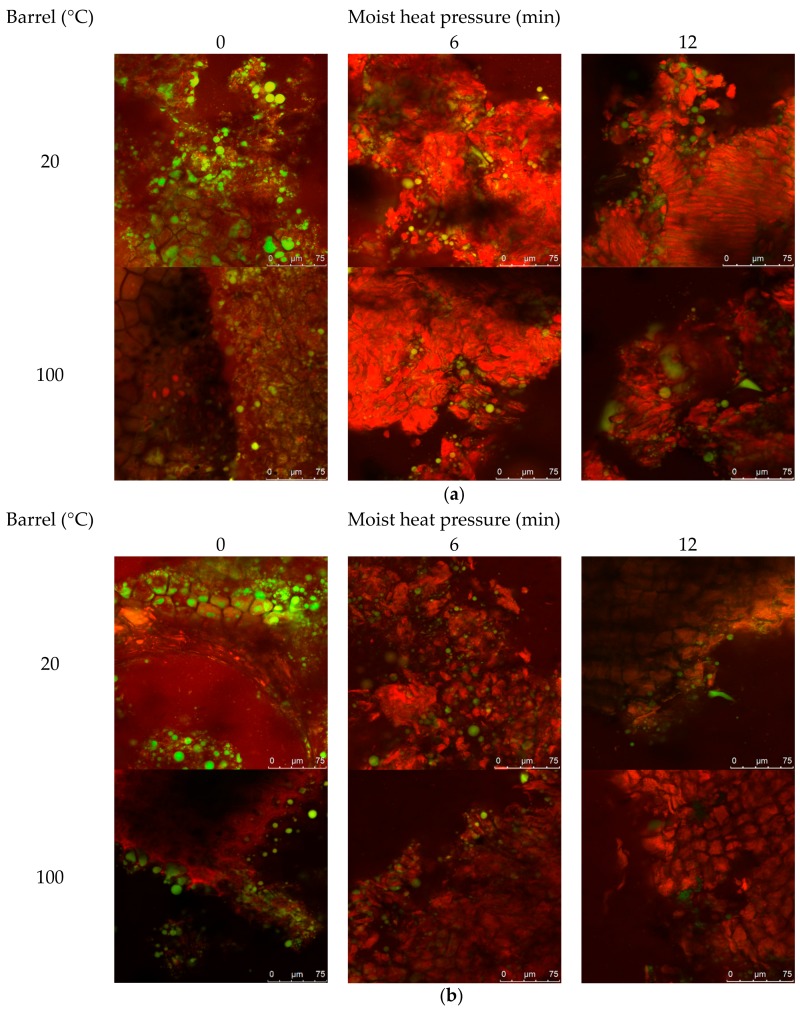
Representative confocal laser scanning micrographs of cold-pressed (20 °C) and expeller (100 °C) canola meal treated with increasing durations of moist heat pressure ((**a**), 0, 6 and 12 min) and proteolytic digestion (**b**). Protein is stained red with Nile Blue dye, and lipid is stained green with Fast Green FCF dye. Scale bars correspond to 100, 75 or 50 µM.

**Figure A2:**
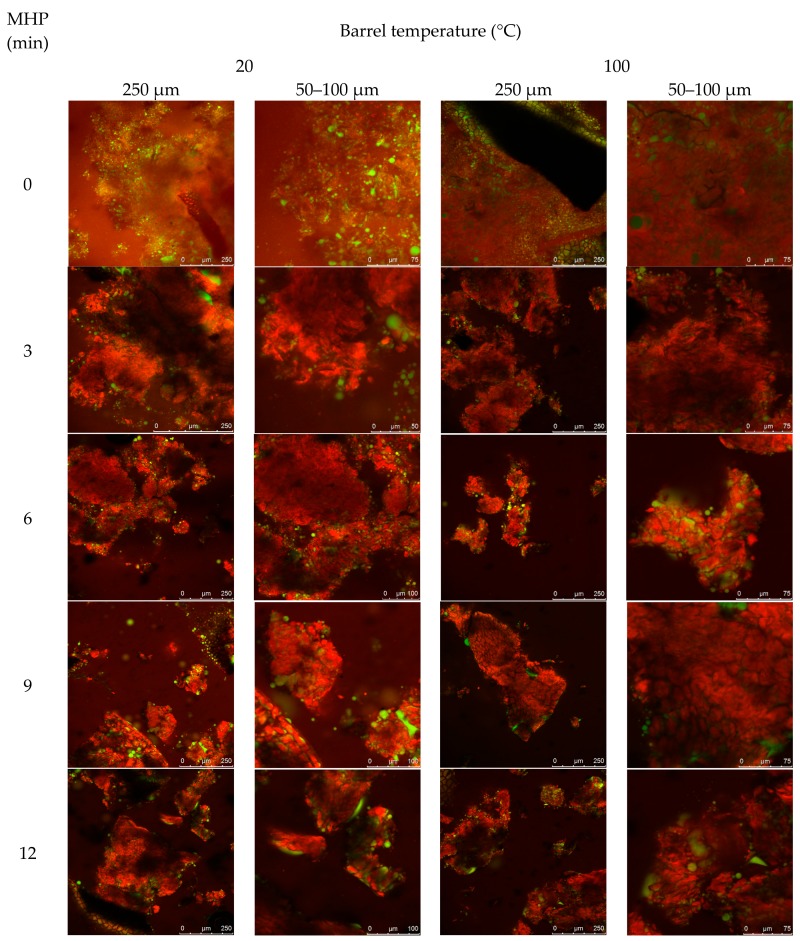
Representative confocal laser scanning micrographs of cold-pressed (20 °C) and expeller (100 °C) canola meal treated with increasing durations of moist heat pressure (MHP, 0–12 min). Protein is stained red with Nile Blue dye, and lipid is stained green with Fast Green FCF dye. Scale bars correspond to 250, 100, 75 or 50 µM.

The effect of MHP duration on canola meal structural organisation, notably protein and lipid, is presented in Figure 2a (and Figure A2 in Appendix A). Untreated meals exhibited intact cotyledon structure, and protein aggregation within and between cellular walls, to produce a heterogeneous matrix with embedded lipid bodies. Barrel associated shearing fractured cell walls surrounding the outer edges of meal flakes, to release lipid bodies (droplets of < ~5 μm). MHP duration constantly produced irregularly sized meal fragments ranging in size from 5–500 μm, that contained dense mats of aggregated protein matrix embedded with < ~15 μm coalesced lipid droplets. The width of internal crevices, created from dense aggregated heterogeneous protein matrix, increased with MHP duration. Residual lipid was observed embedded within the matrix and coalesced within crevices and on the surface of fragments.

**Figure A3:**
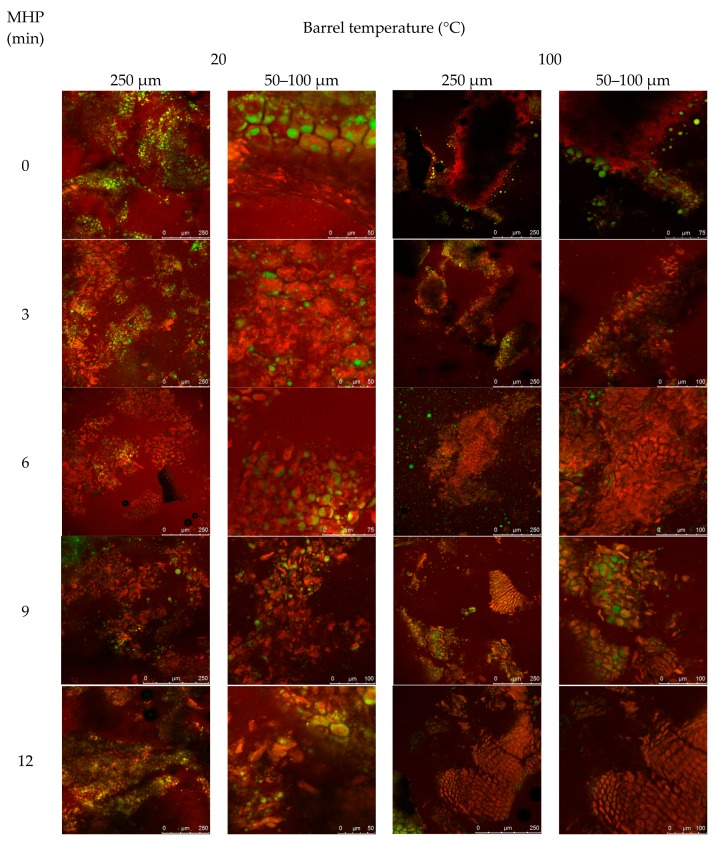
Representative confocal laser scanning micrographs of cold-pressed (20 °C) and expeller (100 °C) canola meal treated with increasing durations of moist heat pressure (MHP, 0–12 min) and proteolytic digestion. Protein is stained red with Nile Blue dye, and lipid is stained green with Fast Green FCF dye. Scale bars correspond to 250, 100, 75 or 50 µM.

The effect of increasing MHP duration on resistance of canola meal to in vitro proteolytic digestion is presented in Figure 2b (and Figure A3). Degradation around sides of the intact cotyledon cellular structure was similar among untreated cold-pressed and expeller canola meals. Fragments of detached protein matrix were observed in both meal types and at all MHP durations. Regardless of MHP duration times, in vitro proteolytic degradation of cellular structure was similar in expelled and cold-pressed meals. After 3 min of MHP, crevices within the aggregated protein matrix widened and after 6 min of MHP, the amount of surface lipid bodies and coalesced lipid droplets within crevices decreased.

### 3.8. Induced Changes in the sSurface Morphology

**Figure 3:**
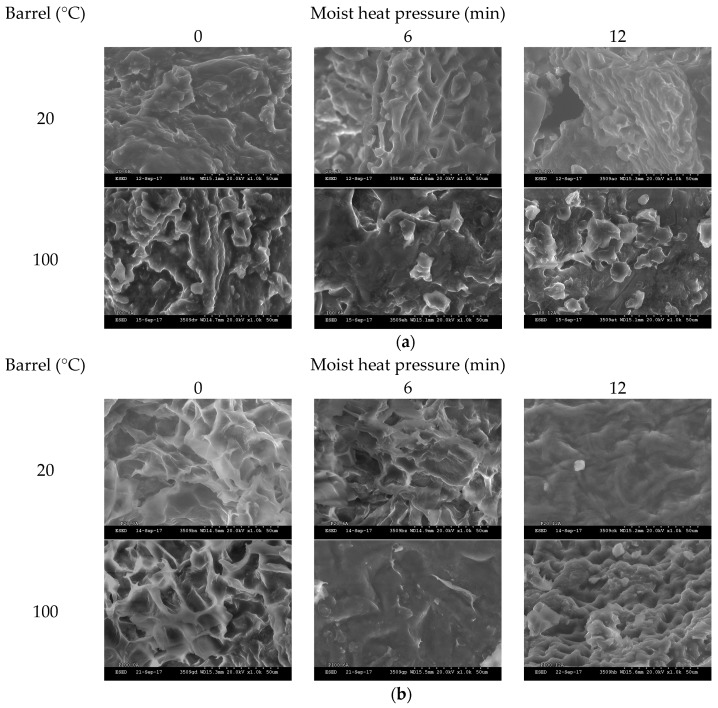
Representative scanning electron photomicrographs of cold-pressed (20 °C) and expeller (100 °C) meal treated with increasing durations of moist heat pressure (**a**, 0, 6 or 12 min) post proteolytic digestion (**b**). Images were taken with ×1.0 k resolution.

**Figure A4:**
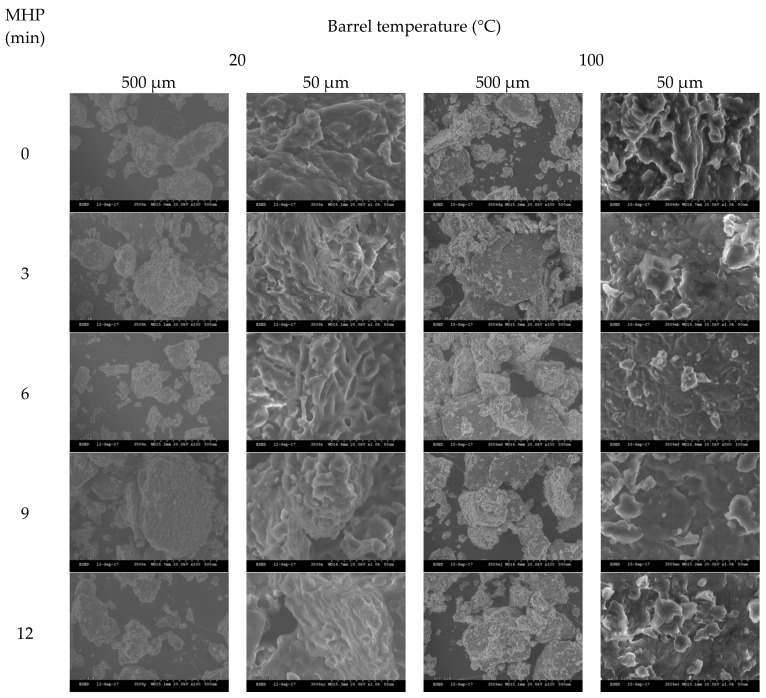
Representative scanning electron photomicrographs of cold-pressed (20 °C) and expeller (100 °C) meal treated with increasing durations of moist heat pressure (MHP, 0–12 min). Images were taken with ×1.0 k and ×0.1 k resolution.

**Figure A5:**
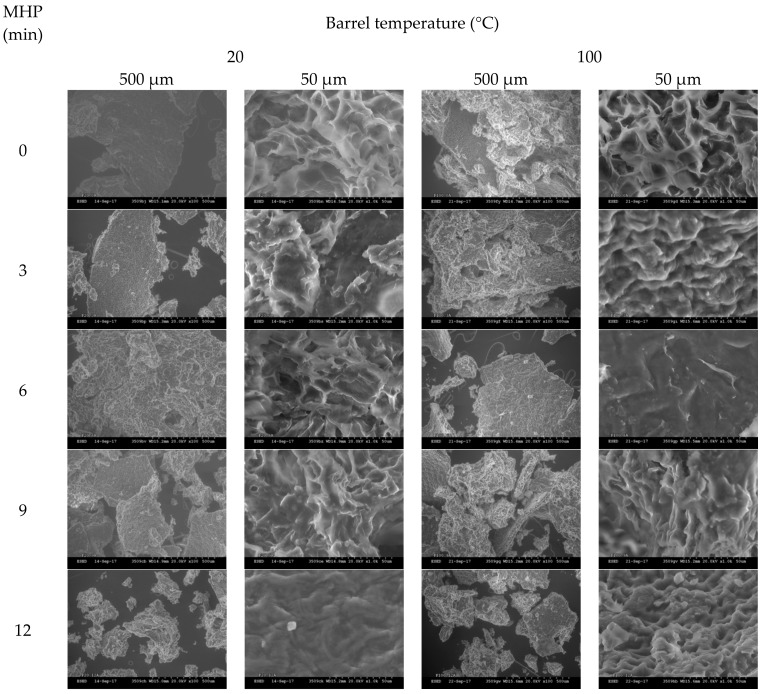
Representative scanning electron photomicrographs of cold-pressed (20 °C) and expeller (100 °C) meal treated with increasing durations of moist heat pressure (MHP, 0–12 min) post proteolytic digestion. Images were taken with ×1.0 k and ×0.1 k resolution.

The effects of increasing MHP duration on meal surface morphology, utilising SEM, are presented in Figure 3a (and Figure A4). At MHP durations of 0 and 3 min, intact, irregular, and complex surface and fragment structures were observed, and at 6, 9 and 12 min the surface of meal became more round and flat. After in vitro proteolytic digestion (see Figure 3b and Figure A5), micrographs revealed surface structures at 9 and 12 min were more intact than at shorter MHP durations.

## 4. Discussion

The cold-pressed and expeller canola meals had similar carbohydrate (15%, [9]), CP, DM, and lipid contents to previously published canola and rapeseed meal values (25.2–38.2% CP, 88.3–96.1% DM, 8.5–17.0% lipid [7,8,41,42,43]). Differences between cold-pressed and expeller meal types were previously reported [8], whereby cold-pressed meal (expelled at 60 °C) contained less DM (91.7 vs. 95.3%) and CP (30.6 vs. 36.1%) and more lipid (17.8 vs. 11.6%) than expeller canola meal (barrel dry heat 98–112 °C). Increased lipid content in cold-pressed and expeller canola meals contributes to greater energy values; thus, monitoring of such is important for the correct formulation of livestock feeds [9]. A reduction of CP at higher temperatures and for prolonged MHP duration suggests increased retention of CP in extracted oil (thereby decreasing the CP content of the meal), or the degradation of thermolabile proteins [44].

While no association of lipid with dry heat was observed, decreased lipid content in rapeseed meal expelled at higher temperatures (104, 112, vs. 121 °C) was previously reported [45]. This study found MHP decreased (*p* < 0.05) DM and had no impact on CP contents of expeller meals. A negative association of DM content with MHP duration suggests the introduction of moisture from steam during the autoclaving process. Moisture reduction by heat evaporation is also known to further catalyse the Maillard browning reaction [46]. Prolonged MHP treatment (120 °C, 1 h) of canola seed was previously reported to not impact (*p* > 0.05) CP, DM, lipid, or carbohydrate (non-treatment 25.2%, 94.9%, 41.8%, 29.0% vs. autoclave treated 25.0%, 94.9%, 44.6%, 26.4%) [25].

The NDF, ADF, NDICP, and ADICP values were similar to values published for canola meal by the National Research Council [47] and DairyOne [48]. A variance of ADF in ground canola seed before and after dry heat (120 °C, 1 h) was similarly reported [25], whereby MHP (120 °C, 1 h) increased (*p* < 0.05) NDICP and NDF, while having no impact on ADF and ADICP. In comparison, this study observed a positive association (*r_s_* = 0.81, *p* < 0.05) of MHP duration with ADICP in the cold-pressed canola meal. The NDICP fraction forms an essential part of RUP [49], while increases in NPN (Fraction A) suggest MHP induces production of instantaneously solubilised peptides [47].

Protein solubility in 0.5% KOH and BP-buffer decreased (*p* < 0.05) with the application of dry heat and reduced (*p* < 0.01) with MHP duration, to imply dry heating disrupted bonds involved in the formation and maintenance of the protein structure to induce protein denaturation [50]. Reduced protein solubility in expeller relative to cold-pressed rapeseed meal (protein solubility in 0.5% KOH, 59.8% vs. 88.3–90.9%, protein solubility in borate, 39.9% vs. 86.8–87.2%) was previously reported [43]. Dry heating (at 100 °C) is postulated to induce denaturation of cruciferin (12S globulin), a major storage protein in canola with an endothermic temperature of 91 °C [51] that accounts for 60% of the total protein in mature seeds [52]. According to soluble protein classifications [53], non-treated cold-pressed meals, and expeller meals treated with MHP for 3 min were very well processed and of high nutritional value (55–60% solubility), whereas MHP treatment cold-pressed meals and expeller meals treated for 0, 6, 9 and 12 min with MHP were overprocessed and declined in nutritional value (<45% solubility). A strong negative relationship of BP-buffer protein solubility with MHP duration implied MHP induces the formation of insoluble protein complexes. Thermal denaturation was found to reduce protein solubility [54]. Furthermore, application of pressure is known to affect quaternary structure by inducing dissociation followed by aggregation of the sub-units or precipitation [55].

Effective CP degradability values of non-treatment cold-pressed (16.8) and expeller (17.3) canola meals were similar (17.8–30.3%, [56]) and higher than other studies (10.8%, [57]) evaluating rapeseed meal. There was no significant difference in effective CP degradability values among non-treatment cold-pressed and expeller canola meals. In contrast, heat during the expelling process (unlike cold press) was found to induce the formation of insoluble peptide chain and carbohydrate complexes, and lower susceptibility to ruminal degradation [10]. The application of MHP (15 min, 117 kPa, 127 °C) to canola meal was similarly reported to considerably decrease levels of in situ N disappearance (69.9 vs. 25.6%) in the rumen of Holstein steers [13]. A strong positive association (*p* < 0.01) of effective CP degradability with MHP duration suggested the formation of insoluble and proteolytic enzyme resistant complexes under MHP conditions. Heating of meal is theorised to favour bypassing of un-denatured protein through the rumen to the lower gastrointestinal tract by promoting protein denaturation and reducing solubility [58]. The IVCPD values for non-treatment cold-pressed (14.1%) and expeller (14.0%) canola meals were similar and agreed with a report for ground canola seed (9.94%) and presscake (16.4%) [59]. Prolonged MHP duration did not impact IVCPD of canola meal and suggests treatment for less than 12 min does not reduce available protein by inducing the formation of insoluble protein complexes with irreversible bonds [60].

Compared to previous reports for rapeseed meal [49,56], lower A, B2, and C, and higher B1 and B3 protein fractions were present in the non-treatment cold-pressed and expeller canola meals. This suggests less immediately (A fraction) and more rapidly (B1 fraction) protein would be solubilised in the rumen; while lower C fraction (unavailable protein) may contribute to an overall increase in AA available post-ruminally [33]. In this study, dry heating (100 °C) was shown to decrease rapidly solubilised (B1 fraction) protein, which implied the formation of insoluble protein complexes with irreversible bonds [60]. Prolonged dry heating (125 °C, 20 min) of expeller canola meal was previously reported to reduce rumen degradability [12], and at 125 °C for 10 min to decrease ruminal CP disappearance without compromising intestinal digestibility [11].

Moshtaghi Nia et al. [13] reported prolonged MHP (117 kPa, 127 °C, 15 and 30 min) treatment of canola meal induced partial protein denaturation and decreased ruminal protein degradability; and, at a duration of 30 min increased the post-ruminal supply of AA for digestion in the small intestine [14]. This study showed at shorter durations of 6 min, rapidly degradable (B1 fraction) protein converted into intermediately digested (B2 fraction) protein, and at 12 min slowly degradable (B3 fraction) protein formed. The timing of these changes corresponds to decreases in protein degradability. Khan et al. [61] previously reported that MHP treatment (120 °C, 60 min) of *Camelina* seeds decreased A and B1 fractions, increased B2 and B3 fractions, and was associated with an increase in in situ RUP. These results showed that MHP at shorter durations significantly changed protein fractions, protein solubility and proteolytic resistance.

The colorimetry values of the canola meals were similar to an earlier report [62]. Dry heat was previously reported to negatively impact lightness, a known indicator of non-enzymatic browning through xylose-glycine and carbonyl-protein reactions and late-MRP formation [63]. MHP duration decreased canola meal whiteness (*p* < 0.05) and yellowness (*p* < 0.01), and increased (*p* < 0.01) redness, BI, and ∆*E*. These results imply MHP induces formation of blue pigments (b*, early MRP) [63], reduces intermediate MRPs (a*) [64], and induces non-enzymatic browning (BI, late MRPs) [63], or protein-polyphenolic reactions [65], likely contributing to the observed darkening (L*), where the degree of colour change increased with MHP duration time (∆*E*). A darker meal is reported to be of beneficial quality for dairy cattle [66]. Early reactions triggering colour change have been observed to impact AA digestibility in monogastrics (poultry) [65]; final Maillard reactions were noted to result in decomposition of involved AAs, where early and advanced reaction products were unavailable for digestion. Further research is necessary to investigate the impact on specific AA in ruminal undegradable protein as the balance of absorbed AA impacts directly on animal production parameters in ruminants.

The pH of meal suspensions was more basic than a database value (5.2, *n* = 4) [48]. Application of MHP increased the pH of the meals, most likely due to an introduction of moisture from steam. With prolonged MHP the pH decreased, likely from intermediate-MRP protein-sugar covalent bond formation. Decreases in pH have been theorised to be associated with Maillard reactions involving amino modification of acid residues by the covalent attachment of reducing sugars in the meal leading to the formation of formic and acetic acids [20]. Reducing sugars are degraded into these products when heated in the presence of protein during the Maillard reaction [67]. A decrease in pH as a function of heating time in controlled (2% porcine plasma) protein-reducing sugar (glucose, fructose and galactose) systems was previously reported [68]. A reduction (*p* < 0.01) of Abs_294nm_ and increase (*p* < 0.05) in darkness and BI (*p* < 0.01) implied MHP further progressed Maillard reactions from intermediate to late, whereas an increase in dry heat increased (*p* < 0.05) Abs_294nm_, implying dry heat progressed early Maillard reactions to intermediate reactions. Induction of Maillard reactions in canola meal processed at a barrel dry heat temperature of 105 °C was similarly reported [65]. Higher UV absorbance was reported as proportional to the presence of open-chain forms of glucose [69]. In consideration of all meals studied, strong associations (*p* < 0.05) of a* (redness) and BI with soluble CP (*r*^2^ = −0.84, −0.85), and effective CP degradability (*r*^2^ = 0.89, 0.91), respectively, were observed. These results imply CIE measures have potential as predictors of soluble CP and effective CP degradability in expeller and cold-pressed canola meals. This is key information to consider when formulating production diets for use in ruminant livestock industries.

The positive correlation of *So* and MHP duration in expeller meal suggests induction of protein unfolding and denaturation [70] and aggregation [71]. Heat treatment of rapeseed napin was found to induce irreversible changes in *So* at a moderate temperature (90 °C) [72]. In this study, observed increases in protein hydrophobicity suggest the occurrence of protein folding events promoting surface hydrophilic conformation, turning of side-chains outwards as with protein unfolding, loss of secondary and tertiary structure, scrambling of disulfide bonds, and formation of irreversible protein aggregates [71,73].

Increasing (*p* < 0.01) dry heat temperature had little impact on molecular protein structure characteristics, except for constricting the amide II region. Elsewhere, prolonged dry heating (120 °C, 1 h) of ground canola seed was found to positively increase (*p* < 0.05) the α-helix-β-sheet ratio, whereas moist heat pressure (120 °C, 1 h) impacted amide I and II regions [25]. Prior research found β-sheet height is a predictor of intestinal digestible in situ RUP (*r*^2^ = 0.83) and total digestible CP (*r*^2^ = 0.81) [23]. This study found an association (*r*^2^ = −0.40, *p* < 0.05) of β-sheet with effective CP degradability, unlike (*r*^2^ = 0.04, *p* > 0.05) the β-sheet with IVCPD.

The molecular structure of cold-pressed meal was more receptive to the effects of MHP than that of expeller meal. In cold-pressed meal, MHP duration constricted amide I and α-helix height, the latter reducing the ratio of the α-helix-β-sheet. Similar impacts of MHP treatment (120 °C, 1 h) on the ratio of the α-helix-β-sheet and amide I in ground canola seed were reported [25]. In cold-pressed meal, unlike expeller, associations (*p* < 0.05) of α-helix and the ratio of α-helix-β-sheet with KOH solubility (*r*^2^ = −0.80, −0.84), soluble CP (*r*^2^ = 0.71, 0.90), and protein degradability (*r*^2^ = −0.73, −0.81), respectively, were observed. These results imply changes in the α-helix and the ratio of the α-helix-β-sheet are likely contributing to protein solubility and digestibility characteristics of cold-pressed canola meal. The ratio of α-helix to β-sheet was found to positively correlate with in situ CP degradation and negatively correlate with the in situ intestinal digestibility of RUP [25]. Previously, MHP and dry heat were reported to have little impact on lipid molecular structure in ground canola seed [39].

In this study, MHP treatment induced changes (*p* < 0.05) in LCCE, CH_2_ and CH_3_ symmetric, and CH_2_ asymmetric regions in cold-pressed canola meal. In addition, dry heat induced changes (*p* < 0.05) in CH_2AS_, CH_3S_, and CH_2S_ regions, and the ratio of CH_3_:CH_2S_. These results imply both processing and MHP treatment can impact lipid molecular structure of canola meal.

Native gel electrophoresis affirmed cruciferin and napin solubility reduced with increasing MHP duration. Progressive temperature-induced changes in the structure of cruciferin were previously reported [70]. These observations imply MHP induces the formation of irreversible bonds and insoluble high MW protein aggregates in canola meal, which may impact the ability to extract protein. Smearing was reported to be indicative of heat-induced modifications of napin at 0–6 min MHP duration [70]. Similar total and water-soluble protein SDS-PAGE polypeptide bands were reported for *Brassica napus* meal [51,74]. In non-reduced conditions, typical storage protein polypeptide bandings were observed for 11S globulin (cruciferin), i.e., trimer (~177), dimer (~118) ([70]), procruciferin subunits (73.5, 76.8), α- (26.7, 28.5, 37.1), β- (18.3, 21.1, 22.9) [75], and monomers with intact disulfide linkages (41–55) [70]; this was also observed for 2S albumin (napin) i.e., dimer (27.5) and monomer (14) [51].

Under reduced conditions, the involvement of disulfide bonds was suggested by the disappearance of polypeptides for cruciferin (i.e., trimers, dimers, and monomers with intact disulfide bands (41–55)), as well as for napin (14), in addition to the associated formation of polypeptide bands for cruciferin (i.e., 9.6–32.0 range) and for napin (i.e., light 4 and heavy 9 kDa). Polypeptide bands present under non- and reduced conditions at 18–25, 27, or 39, or 41 kDa likely corresponded to known oil binding proteins of oleosins, caleosins, or steroleosins, respectively [76]. Gel electrophoresis analysis of canola meal soluble protein fractions revealed MHP impacted both cruciferin and napin solubility, with the degree of solubility differing depending on dry heat temperature. MHP appeared to hinder protein extractability and reduce the solubility of canola meal polypeptides larger than ~40 kDa. Reductions with increased MHP duration again implied MHP induced the formation of irreversible bonds and insoluble high MW protein aggregates in canola meal, which may impact the ability of the animal to break down and utilise protein. Investigations of the heat-moisture effect on wheat flour similarly reported aggregation of lower-molecular weight proteins, the disappearance of albumins and globulins, and the destruction of AAs including lysine [77]. It has been theorised that dry heating of oilseed denatures the protein matrix surrounding fat droplets, in turn functioning to protect dietary fatty acids from biohydrogenation by ruminal bacteria, and increase the supply of polyunsaturated fat to the small intestine [78]. Studies of specific temperatures and time-points of denaturation and aggregations are complicated by the heterogeneous proteinaceous composition of the meal [72].

Little difference was observed in the microscopic structure, lipid and protein conformation of expeller compared to cold-pressed canola meals. Application of MHP for as little as 3 min induced formation of meal fragments of densely aggregated heterogeneous protein matrix, containing crevices where coalesced lipid droplets resided. These structural changes reaffirm a reduction of soluble protein with application of MHP. Application of MHP reduced in vitro proteolytic degradation of the cellular structure relative to the non-treatment meal, reaffirming observed decreases in in vitro ruminal degradability. Rounding and flattening of surface morphology may have been associated with denaturation of canola meal proteins (83 °C) and napin (109 °C) [51]. Structural resistance to in vitro proteolysis was observed at longer MHP durations and warrants further investigations utilising ruminal fluid to better simulate proteolysis in the rumen.

## 5. Conclusions

This study showed the effectiveness of moist heat pressure treatment at shorter duration times to decrease protein degradability while retaining protein value for ruminant digestion. Barrel dry heat was shown to influence canola meal CP, ADF, soluble CP, rapidly digestible protein, napin solubility, intermediate-MRP formation, amide II constriction, and lipid-related molecular structural regions. Moist heat pressure duration time impacted canola meal DM, NPN, NDICP, CP solubility, solubility of canola meal globulin cruciferin and albumin napin proteins, conversion of rapidly into intermediately to slowly degraded protein, protein degradability, the formation of intermediate to late Maillard reaction products, and amide I region constriction. Moist heat pressure altered canola meal structural characteristics including fragmentation of meal to form dense protein aggregates resistant to in vitro proteolysis, with crevices containing coalesced lipid droplets. These changes may impact on ruminal degradation and supply of protein and AA for dairy cattle milk production. CIE measures of a* and BI demonstrated potential as rapid indicators of soluble CP and protein degradability in canola meal, respectively. To the authors best knowledge this is the first report of the microscopic structure and protein and lipid characteristics of cold-pressed and expeller canola meals treated with moist heat pressure. Further analysis of the interrelationships between processing-induced changes in molecular structure, dietary fatty acid, and milk composition are required.

Findings benefit producers of canola meal by further detailing the effects of moist heat pressure duration and barrel dry heat on general properties, protein degradability, and structural and ruminal digestibility characteristics.

## Figures and Tables

**Table 1:** General chemical and protein degradability characteristics of cold-pressed (20 °C) and expeller (100 °C) canola meals with moist heat pressure treatment for durations of 0, 3, 6, 9 or 12 min.

Characteristic	Barrel	Moist Heat Pressure Duration (min)					SEM	P_MHPD_	P_BT_	P_All_	*r_s_*	*r* ^2^
	(°C)	0	3	6	9	12						
CP (% DM)	20	33.8 ^bc^	34.5 ^ab^	34.3 ^b^	33.5 ^c^	33.7 ^c^	0.11	*	**	*	−0.35 *	0.10
	100	33.2	33.4	33.4	33.8	33.4	0.08	NS			−0.36 *	0.25
DM (% AsIs)	20	92.8 ^ab^	92.1^abc^	92.6 ^bcd^	92.2 ^a^	91.2 ^d^	0.17	**	NS	**	−0.74 *	0.54
	100	93.1 ^a^	93.3 ^a^	92.5 ^ab^	92.6 ^ab^	91.9 ^a^	0.16	**			−0.80 *	0.57
Lipid (% DM)	20	15.9	16.3	15.5	15.9	16.4	0.13	NS	NS	*	0.30	0.15
	100	15.3 ^b^	16.3 ^a^	16.4 ^a^	16.4 ^a^	16.5 ^a^	0.14	*			0.50	0.60
Carbohydrate (% DM)	20	14.9	14.8	15.0	14.9	14.8	0.39	NS	NS	NS	−0.26	0.12
	100	15.0 ^a^	15.0 ^a^	14.7 ^b^	14.8 ^ab^	14.7 ^b^	0.47	*			−0.58 *	0.15
NPN (% DM)	20	0.09 ^b^	0.09 ^b^	0.11 ^a^	0.11 ^a^	0.12 ^a^	0.00	**	NS	NS	0.84 *	0.86
	100	0.10 ^c^	0.11 ^c^	0.12 ^b^	0.12 ^ab^	0.13 ^a^	0.00	**			0.99 *	0.95
ADF (% DM)	20	15.1	15.6	15.9	15.1	15.6	0.13	NS	*	NS	0.25	0.23
	100	16.6	16.1	15.5	15.9	15.7	0.18	NS			−0.52	0.39
NDF (% DM)	20	19.4	19.7	19.9	20.9	22.6	0.44	NS	NS	NS	0.86 *	0.68
	100	19.5	19.9	21.0	21.7	21.6	0.35	NS			0.81 *	0.72
ADICP (% DM)	20	1.34	1.44	1.53	1.59	1.63	0.04	NS	NS	NS	0.81 *	0.67
	100	1.63	1.93	1.71	1.87	1.59	0.07	NS			0.03	0.20
NDICP (% DM)	20	5.37 ^b^	5.92 ^b^	6.29 ^b^	7.39 ^ab^	9.99 ^a^	0.58	**	NS	NS	0.99 *	0.75
	100	5.20 ^b^	5.76 ^b^	6.77 ^ab^	8.80 ^a^	9.11 ^a^	0.54	**			0.94 *	0.89
Soluble protein (% CP)	20	70.0 ^a^	49.5 ^b^	31.1 ^c^	28.4 ^cd^	23.0 ^d^	4.46	**	*	NS	−0.97 *	0.93
	100	69.0 ^a^	45.0 ^b^	32.1 ^c^	28.9 ^cd^	22.1 ^d^	4.48	**			−0.95 *	0.94
Solubility 0.5% KOH (%)	20	55.1 ^a^	41.3 ^b^	35.6 ^c^	24.1 ^d^	20.4 ^d^	3.37	**	*	NS	−0.98 *	0.88
	100	34.7 ^c^	54.9 ^a^	43.6 ^b^	43.9 ^b^	22.7 ^d^	2.95	**			−0.35 *	0.80
ECPD (%CP)	20	16.8 ^d^	26.4 ^c^	34.7 ^b^	37.4 ^b^	43.4 ^a^	2.58	**	NS	*	0.96 *	0.97
	100	17.3 ^d^	29.1 ^c^	35.1 ^b^	39.4 ^ab^	42.9 ^a^	2.45	**			0.97 *	0.96
IVCPD (%)	20	14.1	14.1	14.6	14.7	14.7	0.20	NS	NS	**	0.45	0.22
	100	14.0	13.7	14.1	14.0	13.5	0.17	NS			−0.27	0.09

CP: crude protein; DM: dry matter; NPN: non-protein N; ADF, ADICP: acid-detergent fibre and insoluble CP, respectively; NDF, NDICP: neutral-detergent fibre and insoluble CP, respectively; ECPD: in vitro effective CP degradability; IVCPD: in vitro CP digestibility; MHP: moist heat pressure. Means in rows with unlike superscripts differ (*p* < 0.05). SEM: standard error of mean; *r_s_*: pair-wise Spearman correlation coefficient; *r*^2^: coefficient of determination; P_MHPD_: difference between MHP duration times; P_BT_: difference between barrel temperatures at 0 min of MHP; P_All_: difference between barrel temperatures inclusive of MHP treatment samples; ** *p* < 0.01; * *p* < 0.05; NS: not significant.

**Table 2:** Cornell Net Carbohydrate and Protein System protein fractions of cold-pressed (20 °C) and expeller (100 °C) canola meals with moist heat pressure treatment for durations of 0, 3, 6, 9 or 12 min.

Protein Fraction	Barrel	Moist Heat Pressure Duration (min)					SEM	P_MHPD_	P_BT_	P_All_	*r_s_*	*r* ^2^
	(°C)	0	3	6	9	12						
A	20	0.05 ^c^	0.06 ^c^	0.09 ^b^	0.11 ^b^	0.17 ^a^	0.01	**	*	NS	0.99 *	0.90
	100	0.05 ^b^	0.07 ^b^	0.12 ^b^	0.15 ^a^	0.16 ^a^	0.05	**			0.96 *	0.94
B1	20	69.8 ^a^	53.1 ^b^	41.1 ^c^	34.8 ^c^	23.6 ^d^	5.45	**	**	NS	−0.99 *	0.94
	100	66.9 ^a^	48.5 ^b^	33.4 ^c^	28.5 ^c^	26.2 ^c^	5.13	**			−0.96 *	0.98
B2	20	14.4 ^c^	29.8 ^b^	40.6 ^a^	43.3 ^a^	46.8 ^a^	4.50	**	NS	NS	0.99 *	0.93
	100	17.4 ^b^	34.2 ^a^	46.1 ^a^	45.4 ^a^	46.3 ^a^	3.84	**			0.79 *	0.93
B3	20	11.9 ^b^	13.0 ^b^	13.8 ^b^	17.2 ^b^	24.6 ^a^	1.62	*	NS	NS	0.99 *	0.75
	100	10.8 ^c^	11.5 ^c^	15.2 ^bc^	20.5 ^ab^	22.7 ^a^	1.66	*			0.94 *	0.87
C	20	3.95	4.16	4.46	4.70	4.81	0.13	NS	NS	**	0.86 *	0.70
	100	4.90	5.76	5.14	5.51	4.76	0.21	NS			0.03	0.19

Calculated as previously described [33], using B3 (NDIN − ADIN), and B2 (Total CP − (A + B1 + B3 + C)). A: non-protein N; B1: rapidly degraded true protein; B2: intermediately degraded true protein; B3: slowly degraded true protein; C: undegradable true protein; MHP: moist heat pressure. Means in rows with unlike superscripts differ (*p* < 0.05). SEM: standard error of mean; *r_s_*: pair-wise Spearman correlation coefficient; *r*^2^: coefficient of determination; P_MHPD_: difference between MHP duration times; P_BT_: difference between barrel temperatures at 0 min of MHP; P_All_: difference between barrel temperatures inclusive of MHP treatment samples; ** *p* < 0.01; * *p* < 0.05; NS: not significant.

**Table 3:** Monitoring of Maillard reaction product formation in cold-pressed (20 °C) and expeller (100 °C) canola meals with moist heat pressure treatment for durations of 0, 3, 6, 9 or 12 min.

Characteristic	Barrel (°C)	Moist Heat Pressure Duration (min)					SEM	P_MHPD_	P_BT_	P_All_	*r_s_*	*r* ^2^
		0	3	6	9	12						
b*	20	17.0 ^ab^	15.6 ^abcd^	13.2 ^bcd^	14.7 ^bd^	13.9 ^d^	0.425	**	NS	NS	−0.54 *	0.58
	100	17.3 ^a^	17.2 ^a^	15.4 ^ab^	13.8 ^b^	13.2 ^b^	0.488	**			−0.90 *	0.80
a*	20	1.56 ^c^	1.87 ^c^	3.12 ^b^	3.53 ^ab^	4.73 ^a^	0.290	**	NS	NS	0.94 *	0.81
	100	1.80 ^b^	2.20 ^b^	2.68 ^a^	3.36 ^a^	3.34 ^a^	0.186	**			0.90 *	0.81
L*	20	54.5 ^a^	54.1 ^abc^	51.3 ^cd^	52.5 ^cd^	51.5 ^d^	0.422	*	NS	*	−0.64 *	0.51
	100	54.7 ^a^	56.6 ^a^	54.6 ^ab^	53.4 ^bc^	52.5 ^c^	0.447	*			−0.69 *	0.55
∆E	20	0.80 ^c^	2.72 ^b^	5.30 ^a^	3.68 ^ab^	5.17 ^ab^	0.470	**	NS	NS	0.73 *	0.73
	100	0.17 ^b^	2.14 ^b^	2.30 ^b^	4.17 ^a^	5.05 ^a^	0.431	**			0.88 *	0.75
Browning index	20	68.2 ^c^	73.4 ^c^	97.0 ^b^	106.6 ^ab^	122.5 ^a^	5.660	**	NS	NS	0.95 *	0.90
	100	73.4 ^d^	81.0 ^cd^	90.0 ^bc^	101.8 ^ab^	102.9 ^a^	3.460	**			0.87 *	0.77
pH	20	6.39 ^d^	6.54 ^a^	6.54 ^a^	6.49 ^b^	6.43 ^c^	0.016	**	NS	NS	0.03	0.83
	100	6.40 ^b^	6.53 ^a^	6.50 ^a^	6.48 ^ab^	6.42 ^b^	0.014	**			0.01	0.67
Intermediate MRP	20	1.61 ^ab^	1.81 ^a^	1.60 ^ab^	1.45 ^bc^	1.22 ^c^	0.059	**	*	NS	−0.74 *	0.72
	100	1.93 ^ab^	1.86 ^ab^	1.20 ^bcd^	1.53 ^cb^	1.34 ^c^	0.084	**			−0.63 *	0.54
*So* (% soluble CP)	20	3.73	4.02	3.55	4.21	5.12	0.385	NS	NS	NS	0.30	0.21
	100	1.94 ^b^	2.62 ^ab^	4.72^a^	4.19 ^a^	4.15 ^a^	0.374	*			0.71 *	0.72

b*: yellowness; a*: redness; L*: whiteness; MRP: Maillard reaction product; ∆*E*: degree of colour change; *So*: surface hydrophobicity; MHP: moist heat pressure. Means in rows with unlike superscripts differ (*p* < 0.05). SEM: standard error of mean; *r_s_*: pair-wise Spearman correlation coefficient; *r*^2^: coefficient of determination; P_MHPD_: difference between MHP duration times; P_BT_: difference between barrel temperatures at 0 min of MHP; P_All_: difference between barrel temperatures inclusive of MHP treatment samples; ** *p* < 0.01; * *p* < 0.05; NS: not significant.

**Table 4:** Changes in protein molecular structure of cold-pressed (20 °C) and expeller (100 °C) canola meals with moist heat pressure treatment for durations of 0, 3, 6, 9 or 12 min.

Characteristic	Barrel	Moist Heat Pressure Duration (min)					SEM	P_MHPD_	P_BT_	P_All_	*r_s_*	*r* ^2^
	(°C)	0	3	6	9	12						
Amide I	20	0.706 ^a^	0.680 ^a^	0.549 ^ab^	0.695 ^a^	0.435 ^b^	0.0450	*	NS	NS	−0.53 *	0.48
	100	0.607 ^abc^	0.670 ^ab^	0.435 ^bc^	0.55 ^bc^	0.501 ^c^	0.0281	*			−0.42	0.22
Amide II	20	0.299 ^a^	0.258 ^ab^	0.237 ^ab^	0.313 ^a^	0.187 ^b^	0.0158	*	**	NS	−0.42	0.19
	100	0.271	0.343	0.247	0.292	0.273	0.0125	NS			−0.15	0.03
α-helix	20	0.013 ^a^	0.014 ^a^	0.011 ^ab^	0.011 ^ab^	0.008 ^b^	0.0007	**	NS	NS	−0.79 *	0.61
	100	0.013	0.011	0.009	0.011	0.010	0.0005	NS			−0.31	0.26
β-sheet	20	0.012 ^ab^	0.014 ^a^	0.012 ^a^	0.013 ^ab^	0.010 ^b^	0.0005	*	NS	**	−0.43	0.41
	100	0.011	0.012	0.008	0.011	0.010	0.0005	NS			−0.34	0.15
AI:AII	20	2.368	2.699	2.301	2.220	2.382	0.2616	NS	NS	NS	−0.30	0.86
	100	2.326	1.954	1.755	1.888	1.826	0.0807	NS			−0.37	0.39
α:β	20	1.094 ^a^	0.962 ^ab^	0.903 ^b^	0.853 ^b^	0.831 ^b^	0.0281	**	NS	**	−0.90 *	0.80
	100	1.153	0.986	1.060	1.095	1.104	0.0290	NS			−0.03	0.12
TPFR	20	1.341	1.380	1.275	1.355	1.200	0.0244	NS	NS	NS	−0.47	0.30
	100	1.297	1.372	1.198	1.286	1.279	0.0224	NS			−0.20	0.06

Attenuated total reflectance-Fourier transform infrared spectrum absorbance units were analysed for protein molecular structure regions, as previously described by Samadi and Yu [39]. The total protein fingerprint region (TPFR) ca. 1714–1480 cm^−1^ included amide I (AI area ca. 1714–1571 cm^−1^), amide II (AII area ca. 1572–1480 cm^−1^), α-helix (α peak centre height at ca. 1652 cm^−1^ with the baseline of ca. 1714–1480 cm^−1^) and β-sheet (β peak centre height at ca. 1630 cm^−1^ with the baseline of ca. 1714–1480 cm^−1^), as well as the ratio of AI-II (AI:AII), and the ratio of α-β (height) (α:β). MHP: moist heat pressure. Means in rows with unlike superscripts differ (*p* < 0.05). SEM: standard error of mean; *r_s_*: pair-wise Spearman correlation coefficient; *r*^2^: coefficient of determination; P_MHPD_: difference between MHP duration times; P_BT_: difference between barrel temperatures at 0 min of MHP; P_All_: difference between barrel temperatures inclusive of MHP treatment samples; ** *p* < 0.01; * *p* < 0.05; NS: not significant.

**Table 5:** Changes in the lipid-related molecular structure of cold-pressed (20 °C) and expeller (100 °C) canola meals with moist heat pressure treatment for durations of 0, 3, 6, 9 or 12 min.

Characteristic	Barrel (°C)	Moist Heat Pressure Duration (min)					SEM	P_MHPD_	P_BT_	P_All_	r_s_	*r* ^2^
		0	3	6	9	12						
LCCE	20	0.000 ^b^	0.000 ^b^	0.000 ^b^	0.000 ^b^	0.002 ^a^	0.0002	*	NS	**	0.62 *	0.56
	100	0.001	0.001	0.002	0.002	0.002	0.0003	NS			0.29	0.15
CH_3AS_	20	0.000	0.000	0.000	0.000	0.000	0.0001	NS	NS	**	0.47	0.49
	100	0.000	0.000	0.001	0.001	0.000	0.0001	NS			0.29	0.21
CH_2AS_	20	0.000 ^b^	0.000 ^b^	0.000 ^b^	0.000 ^b^	0.002 ^a^	0.0003	*	*	**	0.72 *	0.62
	100	0.002	0.002	0.003	0.002	0.002	0.0002	NS			0.22	0.16
CH_3S_	20	0.000 ^b^	0.000 ^b^	0.000 ^b^	0.000 ^b^	0.001 ^a^	0.0001	*	*	**	0.78 *	0.59
	100	0.001	0.001	0.001	0.001	0.001	0.0000	NS			0.44	0.36
CH_2S_	20	0.000 ^b^	0.000 ^b^	0.000 ^b^	0.000 ^b^	0.001 ^a^	0.0002	*	*	**	0.79 *	0.62
	100	0.001	0.001	0.002	0.001	0.001	0.0001	NS			0.09	0.17
CH_3_:CH_2AS_	20	−0.363	−3.330	−0.828	0.998	0.407	0.6815	NS	NS	NS	0.08	0.14
	100	0.464	0.463	0.403	0.433	0.501	0.0226	NS			0.07	0.11
CH_3_:CH_2S_	20	−2.725	−2.688	1.481	12.21	0.830	2.2728	NS	**	NS	0.69 *	0.16
	100	0.798	0.592	0.545	0.576	1.352	0.1483	NS			0.04	0.27

Attenuated total reflectance-Fourier transform infrared spectrum absorbance units were analysed for lipid-related molecular structure regions, as previously described by Samadi and Yu [39]. Regions included the lipid carbonyl C=O ester stretching band (LCCE, baseline ca. 1789–1701 cm^−1^ with peak height ca. 1744 cm^−1^), asymmetric CH_3_ (CH_3A_ ca. 2988–2951 cm^−1^ with peak height centre at ca. 2955 cm^−1^), asymmetric CH_2_ (CH_2A_ ca. 2951–2882 cm^−1^ with peak height centre at ca. 2922 cm^−1^), symmetric CH_3_ (CH_3S_ ca. 2882–2868 cm^−1^ with peak height centre at ca. 2872 cm^−1^), and symmetric CH_2_ (CH_2S_ ca. 2868–2790 cm^−1^ with peak height centre at ca. 2852 cm^−1^). MHP: moist heat pressure. Means in rows with unlike superscripts differ (*p* < 0.05). SEM: standard error of mean; r_s_: pair-wise Spearman correlation coefficient; *r*^2^: coefficient of determination; P_MHPD_: difference between MHP duration times; P_BT_: difference between barrel temperatures at 0 min of MHP; P_All_: difference between barrel temperatures inclusive of MHP treatment samples; ** *p* < 0.01; * *p* < 0.05; NS: not significant.

## Appendix A

**Table A1:** Linear and polynomial equations to describe the effects of increasing barrel dry heat (20 and 100 **°**C) and moist heat pressure duration on general nutritional properties, protein degradability, and protein fractionation characteristics of canola meals.

Characteristic	Barrel Temperature (°C)	Equation
Crude protein (% DM)	20	Y = 34.17086 + 0.05135x
	100	Y = 33.27538 + 0.02366x
Dry matter (% AsIs)	20	Y = 92.59792 − 0.10669x
	100	Y = 93.30686 − 0.10575x
Lipid (% DM)	100	Y = 15.4352 + 0.2516x − 0.014x^2^
Carbohydrate (% DM)	100	Y = 14.94402 − 0.00932x
Soluble protein (% CP)	20	Y = 64.30043 − 3.57516x
	100	Y = 60.06002 − 3.30252x
Solubility 0.5% KOH (%)	20	Y = 52.51116 + 2.37852x
	100	Y = 63.23841 − 5.62208x + 0.56584x^2^
CP Degradability (%CP)	20	Y = 17.12739 + 3.24392x − 0.08598x^2^
	100	Y = 20.4501 + 2.05132x
NPN (% DM)	20	Y = 3.30687 + 0.15978x − 0.00712x^2^
	100	Y = 3.44565 + 0.08882x
NDF (% DM)	20	Y = 18.56099 + 0.34936x
	100	Y = 0.10015 + 0.0025x
ADICP (% DM)	20	Y = 1.31674 + 0.0343x
NDICP (% DM)	20	Y = 4.25795 + 0.49673x
	100	Y = 4.95482 + 0.36212x
A (% CP)	20	Y = 0.0177 + 0.01406x
	100	Y = 0.04959 + 0.0101x
B1 (% CP)	20	Y = 73.68596 − 5.31728x
	100	Y = 67.03255 − 7.40306x + 0.33499x^2^
B2 (% CP)	20	Y = 13.99265 + 3.81137x
	100	Y = 17.70644 + 6.5357x − 0.353x^2^
B3 (% CP)	20	Y = 8.4819 + 1.38412x
	100	Y = 9.56942 + 1.09073x
C (% CP)	20	Y = 3.8218 + 0.10772x

**Table A2:** Linear and polynomial equations to describe the effects of increasing barrel dry heat (20 and 100 °C) and moist heat pressure duration on Maillard reaction product formation and protein and lipid-related molecular structure of canola meals.

Characteristic	Barrel Temperature (°C)	Equation
*b* *	20	Y = 17.04743 − 0.72851x + 0.04063x^2^
	100	Y = 17.688 − 0.38522x
*a* *	20	Y = 1.43867 + 0.26433x
	100	Y = 1.81067 + 0.14733x
*L* *	20	Y = 54.28733 − 0.25033x
	100	Y = 55.92267 − 0.26x
∆*E*	20	Y = 1.59069 + 0.32311x
	100	Y = 1.0045 + 0.32649x
Browning index	20	Y = 65.17286+ 4.72907x
	100	Y = 73.7597 + 2.66263x
pH	20	Y = 6.405 + 0.04527x − 0.00367x^2^
	100	Y = 6.41491 + 0.03355x − 0.00283x^2^
Intermediate-MRP	20	Y = 1.76237 − 0.0379x
	100	Y = 1.87037 − 0.05013x
*So* (% soluble CP)	100	Y = 1.74298 + 0.58586x − 0.03213x^2^
Amide I	20	Y = 0.45823 + 0.02123x
α-helix	20	Y = 0.01398 − 4.34865 × 10^−4^x
AI:AII	20	Y = 0.24981 + 0.67853x − 0.04446x^2^
α:β	20	Y = 1.05539 − 0.02113x
LCCE	20	Y = −4.04408 × 10^−4^ + 1.269 × 10^−4^x
CH_2AS_	20	Y = −3.49607 × 10^−4^ + 1.65815 × 10^−4^x
CH_3S_	20	Y = 1.34042 × 10^−4^ + 3.47025 × 10^−5^x
CH_2S_	20	Y = −3.36526 × 10^−4^ + 9.70012 × 10^−5^x
CH_3_:CH_2S_	20	Y = −3.17236 × 10^−4^ + 0.73354x
